# Development and Implementation of Novel Virtual Triage and Exploration of Attitudes Towards the Potential Use of Artificial Intelligence in the Irritable Bowel Syndrome (IBS) Dietetic Pathway

**DOI:** 10.1111/jhn.70210

**Published:** 2026-02-04

**Authors:** Emma Stennett, Katerina Belogianni, Miranda Lomer

**Affiliations:** ^1^ Department of Nutrition and Dietetics Guy's and St Thomas' NHS Foundation Trust London UK; ^2^ Department of Nutritional Sciences King's College London London UK

**Keywords:** artificial intelligence, digital triage, irritable bowel syndrome

## Abstract

**Background:**

Dietary management is integral to the irritable bowel syndrome (IBS) pathway. Triage facilitates the decision‐making process for the right dietetic intervention; however, telephone triage is time intensive. Digital advances provide an opportunity to target waiting times and clinical capacity. The aim of this work was to develop and implement a novel semi‐automation virtual triage, assess its impact in the IBS pathway and to investigate attitudes towards the use of artificial intelligence (AI) in triage and dietetic healthcare.

**Methods:**

The Consolidated Framework for Implementation Research (CFIR) provided a structure to develop and implement virtual triage into the IBS pathway. A digital triage questionnaire was developed using experience‐based co‐design. The efficacy of virtual triage was compared with telephone triage for waiting times from referral to triage, clinicians' time taken to triage and clinical capacity. Using qualitative interviews, views on AI in virtual triage and the IBS pathway were collected from three patients and two dietitians who had experience of the newly developed virtual triage process. An exploratory survey in seven gastroenterology dietitians was used to assess attitudes and experiences of AI in clinical practice.

**Results:**

A digital questionnaire was developed and embedded into the IBS pathway for virtual triage. Following implementation, 643 patients received virtual triage with 83% completing the digital questionnaire. From telephone triage to virtual triage, mean waiting times reduced from 56.6 days to 17.5 days, mean clinician time to triage decreased from 20 min/patient to 11 min/patient, and clinical capacity increased from 400 to 1000 appointments/year (all *p* < 0.001). Views on AI in healthcare were mixed, and three key themes emerged: potential benefits; concerns about its use and criteria for application.

**Conclusions:**

Virtual triage increases clinical capacity and reduces waiting times without increasing clinician burden. Attitudes towards AI show interest; however, there is a need for validation to determine confidence and acceptability for both clinicians and patients in terms of problem‐solving and healthcare efficiency.

## Summary

- Digital transformation of the triage clinic increased clinical capacity, decreased waiting times from referral to triage and reduced clinician time spent on triage.
- Experience‐based co‐design of service development was vital to ensure innovation is accessible and acceptable for patients.
- Patients and staff attitudes towards AI use in healthcare were generally positive but understandably tentative; however, with a robust testing and validation process it could be a highly beneficial tool to improve patient care.

## Graphical abstract

Digital triage and attitudes towards potential use of artificial intelligence in the dietetic IBS pathway

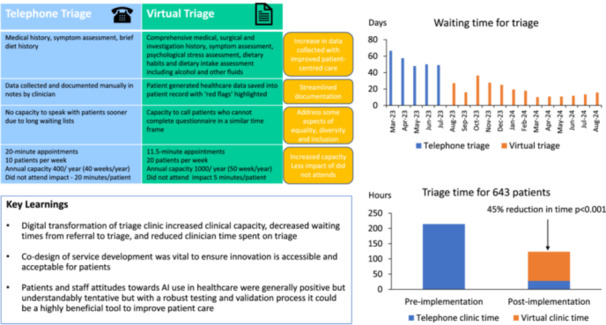

## 1 Introduction

Since the COVID‐19 pandemic, increased waiting times have become a prominent issue in healthcare with the National Health Service (NHS) reporting that 58% of patients wait longer than 18 weeks to receive treatment [1]. The clinical capacity required may be much higher than that available. Healthcare systems provide an opportunity to improve efficiency for innovation. In the last 5 years, the use of digital technology has become commonplace for the delivery of clinical services including telephone and video clinics. Digital technology can improve and update the resources available to reduce waiting times and increase service capacity [2]. Integrating artificial intelligence (AI) alongside digitalisation provides potential opportunities for further improvement in healthcare; however, there are many concerns about where and how it can be used safely and efficiently without carefully planned terms and conditions for use [3]. How well resources are allocated and used to meet the needs and demands of a service defines its clinical efficiency and can directly affect the capacity of a service. Developments to improve clinical efficiency without increased costs are of utmost importance, due to an increase in demand in clinical services predicted with an ageing population and an increase in long‐term conditions [4]. Co‐design is used to develop services, which involves patients and clinicians to jointly make decisions to ensure quality and value of care are maintained, while improving clinical efficiency, clinical outcomes and accessibility such as waiting times [5]. Digitalisation and automation to collect patient data to facilitate triage could be considered the initial step of AI, which specifically refers to computerised systems that perform tasks and cognitive functions (e.g., via algorithms or rules) to process various problems without human intervention [6]. AI can be used in healthcare to facilitate clinical decision‐making, empower clinician–patient interactions and patient‐centred care [7]. With a movement towards a more digital system, harnessing the benefits of AI in healthcare is of interest [8].

Irritable bowel syndrome (IBS) is a disorder of gut–brain interaction and occurs in approximately 5% of the population depending on the assessment criteria used [9, 10]. It is a chronic debilitating disorder characterised by altered bowel habits associated with abdominal pain and bloating, and impairs health‐related quality of life [11]. It negatively impacts direct and indirect healthcare costs with a huge burden on the economy. Direct healthcare costs for the UK are £90–£316/patient/year, while indirect costs are between £400 and £900/patient/year [12].

Many patients with IBS report that dietary components trigger gastrointestinal symptoms; thus, dietary management strategies are central to IBS clinical guidelines [13, 14, 15]. Treatment options include first line (healthy eating for IBS including fibre modification) and second line (low fermentable oligosaccharide, disaccharide, monosaccharide and polyol [FODMAP] diet) dietary approaches. The low FODMAP diet is particularly effective in diarrhoea‐predominant (IBS‐D) or mixed IBS (IBS‐M), while fibre modification is more effective for constipation‐predominant IBS (IBS‐C) [13]. The success of dietary management in the IBS pathway has increased demand for dietetic services [16]. Innovation includes dietitian‐led group education for counselling on the low FODMAP diet [17, 18]. Such group education is not only as clinically effective as traditional one‐to‐one counselling, but it also increases clinical capacity and reduces waiting times for treatment [19, 20]. First‐line and/or constipation dietary counselling is also successfully delivered in dietitian‐led groups [21].

A holistic approach to IBS management is of paramount importance [15], and it is imperative that dietitians are able to explain to patients the magnitude that stress and anxiety may have in the aetiopathogenesis of IBS as a disorder of gut–brain interaction [22]. Tools to measure stress and/or anxiety are useful additions for comprehensive assessments provided by dietitians and the multi‐disciplinary team [23].

Triage enables patients with different IBS subtypes to be directed to the most effective management strategies, particularly where one‐to‐one appointments and group education are offered. Telephone triage provides an opportunity to gather information specifically on symptoms, along with dietary assessment, facilitating the decision‐making process to direct patients to the most appropriate dietetic intervention [18]. However, telephone triage increases appointment burden for both dietitians and patients, lengthens clinician time and extends patients' waiting time for treatment, all of which negatively impact clinical capacity. Furthermore, dietitians report a lack of specialist dietitians and established IBS pathways [24], increasing the number of IBS referrals and creating frustration for patients where access to IBS services has very long waiting times [16]. In addition, dietitians describe that it can be challenging to triage patients, specifically obtaining relevant clinical, symptom and dietary information on the telephone or by post and acknowledge that digitalisation may help [16]. Digital transformation utilising electronic patient platforms to send out online questionnaires enables faster triage [3] and improves clinician experience [25]. The use of questionnaires to triage IBS patients to patient webinars is an example of successful innovation in the IBS pathway [26].

This project aimed to (i) develop a digital triage questionnaire, evaluate and implement its use in a novel semi‐automated virtual triage process in the dietetic IBS pathway and (ii) investigate attitudes towards the use of AI in triage and dietetic healthcare.

## 2 Methods

### 2.1 Study Design

This was a service development and implementation project using the Consolidated Framework for Implementation Research (CFIR) with five main domains: intervention; outer setting; inner setting; individual characteristics and process of implementation (Supporting information S1: Table S1) [27]. It involved digitalisation of the triage process in a dietetic IBS pathway for outpatients referred from primary and secondary care to a specialist NHS gastroenterology dietetic service. Data were collected for patients vetted for the IBS pathway pre‐implementation using telephone triage (March to July 2023) and post‐implementation using virtual triage (August 2023 to July 2024). It also explored attitudes towards the use of AI in healthcare using a small‐scale exploratory survey and qualitative approach to identify whether further innovation to develop an AI‐powered decision‐making questionnaire was acceptable. The project did not require ethical approval according to the national health research authority tool, and it was registered as a service evaluation in accordance with local quality and assurance compliance (16822).

### 2.2 Intervention Development

#### 2.2.1 Referral Vetting on Entry to the IBS Pathway

Dietitians vetted the suitability of referrals by reviewing relevant investigations in accordance with local and national guidelines, for example, negative coeliac screen, faecal calprotectin < 50μg/g in patients with diarrhoea [14]. Where details of relevant investigations were missing, referrals were rejected back to the referrer requesting the details and if necessary, the relevant investigations. Referrals with information indicating patients were unsuitable for group education were offered one‐to‐one appointments at the point of vetting. These included atypical symptoms, weight loss, history of eating disorder or disordered eating, nutritional concerns or medical history unrelated to IBS needing a more personalised approach, limited comprehension of the English language, learning disability or those who specified a preference for a one‐to‐one appointment as previously reported [18].

Patients suitable for triage were provided with an electronic symptom questionnaire containing the global symptom question, the gastrointestinal symptom rating scale (GSRS) [28] and the Bristol Stool Form Scale assessing stool frequency and consistency [29] to complete before the telephone triage appointment (Supporting information S1: Table S2). The global symptom question evaluated current relief from gut symptoms by asking a single question with a dichotomous ‘yes/no' answer [30]. The GSRS assessed symptom severity and frequency using a 4‐point Likert scale [28].

#### 2.2.2 Telephone Triage (Pre‐Implementation Service)

During the telephone triage appointment, patients were asked about past medical history, investigations related to IBS, history and severity of IBS symptoms, dietary intake and weight history (Supporting information S1: Table S2). At the end of the appointment, outcomes and next steps were discussed with patients. Triage outcomes included patients being offered one of the following: (1) patient group education, which delivered either first‐ or second‐line advice (2) one‐to‐one appointments with a dietitian for patients who were unsuitable for group education (3) discharge from the service for patients whose symptoms had resolved or if the patient no longer needed or wanted to proceed with dietary management.

Triage involved a 20‐min telephone appointment between each patient and a specialist gastroenterology dietitian/dietetic assistant. It took place as a weekly 3.5‐h clinic in one dietitian/dietetic assistant's job plan for 40 weeks per year enabling 400 patients per year to be triaged. The remaining 12 weeks, the telephone triage appointments were cancelled for periods of leave (i.e. annual leave, study leave, bank holidays and sick leave).

#### 2.2.3 Virtual Triage (Post‐Implementation)

##### 2.2.3.1 Development of the Digital Questionnaire

Patients who had experienced telephone triage as part of the dietetic IBS pathway between July 2021 and July 2023, who had consented to being contacted about participating in service development, were invited by email to attend an online focus group. Experience‐based co‐design (EBCD) was used to adapt a basic symptom questionnaire to a comprehensive tool for use in a novel virtual triage clinic as part of the IBS pathway.

Patients who agreed to take part were sent an initial draft of the digital questionnaire to review at least 1 week before their focus group. Patients who attended the focus group were asked to provide feedback on the content, question wording, comprehension, flow and ease of completion of the questionnaire. They were also asked about their attitudes towards the novel virtual triage compared with the telephone triage. Patients were compensated for their time according to national guidance [31].

##### 2.2.3.2 Questionnaire Items

The digital questionnaire included questions as part of the standard nutritional assessment process [32], that is, patient medical and surgical history, investigations, family history, history of food allergy, dietary habits, history of eating disorder/disordered eating. It also included IBS‐related questions on gut symptoms. Gut symptoms were assessed via the global symptom question, GSRS [28], the IBS‐SSS [33] and the Bristol Stool Form Scale assessing stool frequency and consistency [29]. The IBS‐SSS score assesses symptom severity with total scores ranging from 0 to 500, with scores above 300 indicating severe symptoms [33].

Questions on psychological stress and the impact of IBS on daily life were added to identify lifestyle symptom triggers and facilitate holistic interventional approaches. Psychological stress was assessed via the Perceived Stress Scale (PSS) [28], which included 10 questions about feelings and thoughts during the last month with 5‐point Likert‐scale responses [34]. Scores 0–13 indicate low, 14–26 indicate moderate and 27–40 indicate high stress [35]. The Work and Social Adjustment Scale (WSAS) [36] was used to assess the impact of IBS on daily life. The WSAS included five questions to assess mental health difficulties on individuals' ability to function in terms of work, home management, social leisure, private leisure and personal or family relationships. Scores range from 0 to 40. Scores below 10 indicate low, 10–20 indicate moderate and above 20 moderately severe functional impairment [36].

Introduction and outcome (i.e., next steps) sections were also included in the questionnaire to provide further information on the virtual triage process (Supporting information S1: Table S2). Licence agreements were made with the Rome Foundation for the use of the IBS Symptom Severity Score (IBS‐SSS) and Bristol Stool Form Scale for use within the questionnaire.

##### 2.2.3.3 Automation Features

A sliding scale from 0 to 10 was built into five questions for the IBS‐SSS (i.e., abdominal pain severity, days of pain, abdominal distension/bloating severity, satisfaction with bowel functioning and interference of IBS with life in general). Total scores for IBS‐SSS, PSS and WSAS were automated. Specific question responses were set up in ‘bold text' to alert the dietitian of potential clinical concerns during triage, for example, co‐morbidities, unexplained weight loss, blood in stool, unexplained anaemia, history of disordered eating or an eating disorder and scores for IBS‐SSS > 300, PSS > 27 and WSAS > 20.

##### 2.2.3.4 Virtual Triage Implementation

The digital questionnaire was embedded into an electronic patient record system, and a novel virtual triage clinic was set up. Virtual triage enabled dietitians to review the completed questionnaires, decide triage outcomes and facilitate patient‐centred care in the upcoming dietary education appointment (see Supporting information Box S1 for details).

The outcome of virtual triage was provided to patients via text and/or letter. The outcome decisions were one of the following: (1) one‐to‐one appointment, (2) online group education on: first‐line dietary advice for IBS, dietary advice for constipation or the low FODMAP diet or (3) discharge from service for patients no longer needing dietary management. Patients who did not complete the digital questionnaire following all reminders were discharged from the service with an opportunity to rebook within 2 weeks if they still needed dietary management.

The virtual triage clinic was initially piloted and led by a project dietitian for 8 months (August 2023 to March 2024) before being adopted into the IBS pathway in April 2024 as part of standard operating procedures.

During August 2023, the time taken to triage each patient was recorded to determine how much time would be needed for the weekly virtual triage clinic. It resulted in setting up a weekly 3.5‐h virtual clinic for 20 patients. 10 min was allocated to each patient allowing sufficient time for telephone calls to patients where needed as described above. The virtual triage clinic was set up as a shared service for two gastroenterology dietitian job plans on alternate weeks with cover plans in place to ensure the clinic was available for 50 weeks per year to provide 1000 virtual clinic appointments per year. Appointment booking throughout the project was undertaken by the administrative team, and the time taken for this was not accounted for. Discussions between dietitians and the administration team agreed that administration time for appointment booking was not going to change due to digital transformation of triage.

External to this project, a planned update of the hospital electronic patient record took place in October 2023 to a single system called EPIC Healthcare (EPIC Systems Corporation, Verona, Wisconsin, USA) (see Supporting information Box S2 for details).

#### 2.2.4 Outcomes

Patient demographics, waiting times in days from referral to triage, clinician time, in minutes, to complete triage, triage outcomes (one‐to‐one appointment/online group education/discharge from service) for all referred patients and clinical capacity were recorded for the telephone and virtual triage clinics during the project. Clinical outcomes were not reported as part of this project due to the launch of EPIC and associated delays beyond our control in setting up digital questionnaires for follow‐up.

### 2.3 Use of Artificial Intelligence (AI) in Healthcare

#### 2.3.1 Dietitian Survey

In September 2024, dietitians from the specialist NHS gastroenterology dietetic service were invited to complete a short electronic questionnaire on their attitudes and experience towards using AI in clinical practice. The survey questions were developed from topics relevant to the IBS pathway and focused on attitudes to vetting and triage in healthcare, and refined on discussion with the research team. The survey consisted of 10 questions with responses collected using Likert scales, multiple select answers and free‐text answers. There was no scoring system for the responses.

#### 2.3.2 Semi‐Structured Interviews

Patients with IBS who had experienced virtual triage in September 2024 as part of the new dietetic IBS pathway and consented to being contacted about participating in service development were invited by email to provide details of their experience and attitudes on the use of AI in healthcare in one‐to‐one online interviews with the project dietitian, and three patients took part. Two specialist gastroenterology dietitians who were involved in the virtual triage clinic were interviewed to further explore their attitudes towards the use of AI in October 2024. The interviews focused on the potential integration of AI into clinical practice and its application in the referral vetting and triage process, and, in particular, where automation had already been integrated into the virtual triage process (see above).

The semi‐structured interviews were based on similar topics with 14 open‐ended questions, some with further probing questions enabling deeper discussion from the patients' and clinicians' perspective (Supporting Information S1: Table S3).

#### 2.3.3 AI Outcomes

There was no pilot testing of the questions for the survey or the interviews. The intention of the survey and interviews was to explore attitudes towards the use of AI in healthcare and to ascertain acceptability on its use in vetting and triage, particularly following the experience of virtual triage.

### 2.4 Data Collection Analysis

Analysis of quantitative data was carried out using SPSS (SPSS version 29, IBM corporation), where appropriate and an unpaired *t*‐test was used for comparisons of continuous data, and chi‐squared was used for comparisons of categorical data. Statistical significance was reported where *p* < 0.05.

Qualitative data from focus groups were collected for the digital questionnaire. Two clinicians were present at each session to facilitate the discussion and transcription. One clinician was leading on the focus group, and one clinician was observing the process and taking notes. Each focus group lasted for 60 min.

Qualitative data were collected via Microsoft Teams (Version 24165.1406.2974. 9471. Microsoft; 2024) and all sessions were recorded and transcribed following intelligent transcription by editing out fillers and repeated words.

Telephone/video interviews with patients and dietitians to gain insight into attitudes towards AI were undertaken by one clinician using thematic analysis to identify themes and patterns after applying a combination of deductive and inductive approaches to manual coding. This captured perceptions and experiences of AI and additional thoughts that were expressed during interviews [37]. Transcription notes on quotations were taken, and the feedback was organised manually into themes based on the interview questions. Each interview lasted between 15 and 30 min.

## 3 Results

Invitations to develop the questionnaire were sent to 217 patients, who had consented to be contacted, 30 patients expressed an interest in taking part in EBCD and, of those, 14 patients (mean age 53 years [standard deviation (SD) 16], 2 (14%) male) attended focus groups to develop the digital triage questionnaire (see Table 1 for participants' demographics). Main comments for improvement included: a need for clarity on the wording of questions, more detail to explain the purpose of the questionnaire in the introduction, a clear outcome section, space for patients to add more details on their symptoms, provide reasoning for including some questions (e.g. PSS, WSAS and alcohol consumption) and the usability of the online form. Overall feedback was positive with most patients identifying benefits for using a digital questionnaire as part of virtual triage instead of telephone triage (Supporting information S1: Table S4).

**Table 1:** Participant demographics.

Demographics	Questionnaire development patients (*n* = 14)	Provided opinions on artificial intelligence	
		Patients (*n* = 3)	Dietitians (*n* = 8)^a^
Age (mean [SD]) years	53 [16]	33 [2]	36 [10]
Male *n* (%)	2 (14%)	1 (33%)	0
Ethnicity *n* (%)			
White British	6 (43%)	1 (33%)	5 (62.5%)
White Irish	0	0	2 (25%)
Black Caribbean	2 (14%)	0	0
White and Asian	0	0	1 (12.5%)
Other white background	4 (29%)	1 (33%)	0
Other Asian background	1 (7%)	0	0
Other mixed background	1 (7%)	0	0
Prefer not to say	0	1 (33%)	0
Highest qualification *n* (%)			
GCSE or equivalent	3 (21%)	0	0
A‐level or equivalent	0	0	0
Bachelor degree	7 (50%)	2 (67%)	4 (50%)
Masters degree or doctorate	3 (21%)	1 (33%)	4 (50%)
Apprentice/other professional	1 (7%)	0	0
Employment status *n* (%)			
Part‐time	2 (14%)	0	1 (12.5%)
Full‐time	4 (28%)	1 (33%)	7 (87.5%)
Self‐employed	2 (14%)	0	0
Retired	3 (21%)	0	0
Long‐term sick/disabled	2 (14%)	0	0
Unemployed and actively seeking work	0	1 (33%)	0
Carer/other	1 (7%)	1 (33%)	0

^a^ Two of these dietitians provided feedback to the semi‐structured interviews.

**Figure 1:**
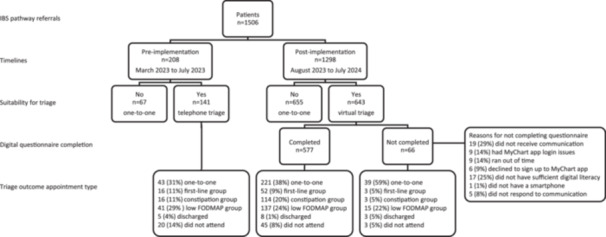
Flow diagram. FODMAP: fermentable oligosaccharide, disaccharide, monosaccharide and polyol. MyChart app patient facing app for interaction with the NHS Trust. Vetting suitability for triage: Pre‐implementation No—not suitable: atypical symptoms, weight loss, history of eating disorder or disordered eating, nutritional concerns or medical history unrelated to IBS needing a more personalised approach, limited comprehension of the English language, learning disability or those who specified a preference for a one‐to‐one appointment [18]. Post‐implementation No—as for pre‐implementation and lack of digital literacy. All patients who were identified to have any clinical concerns had one‐to‐one appointments.

During the service evaluation, 1506 patients were referred to the dietetic IBS pathway (Figure 1), 340 (23%) of those were direct referrals from primary care. Following vetting, 722 patients were unsuitable for telephone triage (*n* = 67) or virtual triage (*n* = 655) and were offered a one‐to‐one appointment. More patients referred from secondary care (631 (42%)) needed one‐to‐one appointments compared with primary care (91 (6%); *p* < 0.001). The remaining 784 were suitable for triage [mean age 39 years (SD 14), 239 (33%) male, primary care 249 (32%)]. Before the implementation of virtual triage, 141 patients were offered telephone triage; 20 patients (14%) did not attend (DNA) and following implementation, 643 patients were offered virtual triage. Of the 643 patients, 532 (83%) completed the digital triage questionnaire, 66 (10%) patients were unable to complete the digital questionnaire and had a telephone triage appointment instead and 48 (7%) patients did not engage with the service, that is, DNA. Reasons for not completing the digital questionnaire are provided in Figure 1.

**Figure 2:**
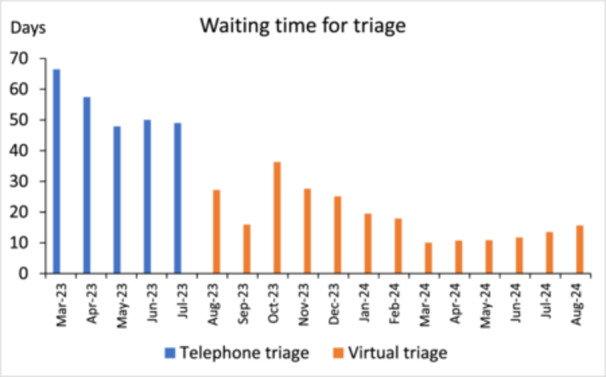
Mean waiting times for triage per month.

The mean waiting time significantly reduced from 56.6 days (SD: 24.1) using telephone triage pre‐implementation, to 17.5 days (SD 12.9) post‐implementation using virtual triage (*p* < 0.001) (Figure 2).

**Figure 3:**
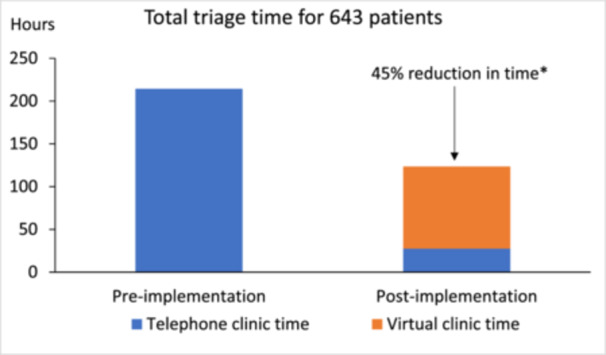
Triage time pre‐ and post‐implementation of virtual triage. *p < 0.001 for the time difference between pre‐implementation and post‐implementation of virtual triage. Virtual triage includes time taken to carry our virtual triage and telephone triage for patients who completed the digital questionnaire enabling virtual triage and telephone triage for patients who could not complete the digital questionnaire within 2 weeks.

The total time taken to triage 643 patients in the virtual triage clinics was 118 h. Of this, 96 h were for 577 patients who completed the digital questionnaire and were assessed using virtual triage, and 22 h were for 66 patients who did not complete the digital questionnaire and/or needed telephone triage. Based on existing data before implementation, it would have taken 214.3 h to triage 643 patients using only telephone triage. Thus, the time taken to triage patients using the virtual triage pathway reduced by 45% as shown in Figure 3. The mean time to triage patients reduced from 20 min/patient for telephone triage to 11 min/patient for virtual triage (*p* < 0.001). The time wasted for DNA reduced from 20 min/patient for telephone triage to less than 5 min for virtual triage.

Clinical capacity for triage increased from 400 appointments per year for telephone triage over 40 weeks to 1000 appointments per year for virtual triage over 50 weeks. This was due to reduced time taken to triage patients allowing more patients to be booked in per week, and changing the virtual triage clinic to a shared service between two dietitians job plans as described in methods.

### 3.1 Attitudes Towards AI

Demographic characteristics for three patients and eight dietitians who participated in the qualitative research are shown in Table 1. From the group of eight dietitians surveyed, no‐one reported AI user‐experience but all were interested to learn more and receive training on how AI may be used in clinical practice. Six (75%) dietitians were familiar with AI technologies being used in healthcare. Seven (88%) dietitians believed that AI could complement dietitians' tasks. There were varying responses on the reliability of using AI tools to assess the nutritional needs of patients with five (63%) responding they would find it very or somewhat reliable, five (63%) would not find it reliable and three (38%) were unsure. The additional responses to the survey on dietitians' views towards the potential uses, benefits and concerns of AI in dietetic practice are provided in Table 2.

**Table 2:** Responses for dietitian survey on artificial intelligence (AI) in dietetic practice.

Areas of dietetics suitable for AI	Dietitians *n* (%) *N* = 8	
Dietary intake tracking	8	(100)
Nutritional deficiency identification	7	(88)
Meal planning	5	(63)
Risk assessment for chronic diseases	5	(63)
Weight management	1	(13)
Recognises portion sizes	1	(13)
Benefits of AI in dietetics		
Faster referral vetting/triaging	8	(100)
Reduce administrative workload	6	(75)
More accurate nutritional analysis	5	(63)
Improve personalisation of diets	5	(63)
Faster assessments	4	(50)
Easier access to data	3	(38)
More accurate and faster nutritional analysis of dietary intake	1	(13)
Semi‐automation of administrative processes will increase time spent effectively elsewhere	1	(13)
Quicker note and letter writing to reduce admin workload	1	(13)
Concerns about using AI in dietetics		
Lack of human empathy/understanding	7	(88)
Decreased patient interaction	5	(63)
Potential for errors or inaccuracies	4	(50)
Over‐reliance on technology	4	(50)
May take away from the purpose of the dietitian	1	(13)
Data privacy concerns	0	

The qualitative analysis of the patient and dietitian interviews identified three main themes (Table 3): *Potential benefits of AI in healthcare; Concerns about the use of AI in healthcare* and *Criteria when applying AI in healthcare*. When asked about their familiarity with AI, patients reported being aware of messaging, completing triage questionnaires and ChatGPT (OpenAI Chat GPT, 2024); however, felt it was still in its infancy in healthcare. Dietitians were not very familiar with the use of AI in their local healthcare setting but expressed an interest to learn more about AI and where it was already being used.

**Table 3:** Themes on artificial intelligence (AI) from patients and dietitians.

Representative patient quote	Representative dietitian quote
**Theme 1: Potential benefits of AI in healthcare**	
*‘It is useful and still feels like you're speaking to someone when using online messaging* *It might help to be seen quicker if care needed urgently’* *‘The speed at which things could be considered and looked at may improve accuracy of assessment and remove human error’* *‘Could improve speed at which care is received and triaged effectively’* *‘AI could help triage to the correct treatment when initially referred and provide some tips until clinician appointment’* *‘AI could help services to be more balanced, clinicians can be biased e.g. doctors have blamed age, circumstances, busy lifestyle, stress on development of my symptoms’* *‘Using AI as a support tool, e.g. having resources of diet, meal plans and recipes could be helpful, particularly for FODMAP diet as it's a daunting process’*	*‘AI has diagnostic potential in gastroenterology’* *‘Could assessment of diet be as good as a dietitian, appreciating the nuances and holistic picture given in triage questionnaire’* *‘AI in referral vetting, could have a role’* *‘If rejection or accept criteria was used for referrals, I would trust this more so than replacing dietitian appointments’* *‘I'm hopeful that AI would help with other non‐patient facing elements of pathway’* *‘Automation of vetting to accept or reject referrals based on specific criteria e.g. catchment area postcode or inclusion of mandatory investigations e.g. coeliac screen result’* *‘AI in triage could highlight red flag symptoms necessitating a one‐to‐one appointment’* *‘AI could place orders and send letters based on the results’* *‘AI could improve efficiency and reduce waiting times so that more time can be spent with patients by reducing clinician time spent on other activities’* *‘It may help processes and time management’* *‘Less time spent documenting patient information’* *‘Ability to give patients more targeted information’* *‘If used wisely to complement/in conjunction with human empathy, clinical reasoning skills and behaviour change skills it could improve accuracy and efficiency (freeing up time for other tasks or other parts of the dietetic consultation) and reducing time spent on* *admin, post consult e.g. good for information gathering/assessment’* *‘It may help to reduce time spent on tasks that do not need to be patient facing’* *‘It could be a useful support tool for reducing time on tasks that are lengthy’* *‘AI could be helpful in reducing time on admin and vetting/triage’* *‘It could be a useful tool for triaging, screening purposes and meal planning’* *‘I think in the right way it could be really positive and a helpful tool, if it reduces workload from tasks such as notes and letters, this could allow more time for other service development/research etc’*
**Theme 2: Concerns about the use of AI in healthcare**	
*‘Don't think it could replace a doctor. Not sure where it would get its information from. Have found errors on ChatGPT when using. Shouldn't be used as replacement for appointments with clinicians’* *‘I don't think it should be used to provide dietetic care or advice, I don't want it to remove in person contact’* *‘I would expect a doctor to read records and come up with a plan, it's more personal and I have years of experience with doctor’* *‘AI could detract from human element and could feel let down by healthcare, patients may need reassurance’* *‘I value human interaction and people's knowledge too much’*	*‘Limited information on triage questionnaires may inhibit decision making’* *‘I don't think AI and questionnaires can replace human interaction and empathy that a dietitian provides to understand patients’ issues’* *‘I think it is important it does not take over/replace what a dietitian offers, and the patient does not feel that they are not listened to’* *‘It may negatively impact on patient experience if there is less patient contact as patients may not feel heard or as well understood if assessments are primarily done through AI’* *‘Exciting but still cautious, I have mixed feelings’* *‘I think more research is needed in this area, currently the evidence is on dietitian‐led services’* *‘Specialist team member is needed to support with implementation’* *‘Wouldn't want it to replace consultations or decision making. Safety perspective, human ability to see holistic picture’* *‘Energy used to store data, will that contribute to carbon footprint in storing data, i.e. the energy used to achieve that’*
**Theme 3: Criteria when applying AI in healthcare**	
*‘I think it could be useful in lower risk elements of healthcare’* *‘Depends on health issues’* *‘I'd want to know that AI has been trained using best practice guidelines, by things that medical professionals would refer to as well’* *‘I'd need transparency over process of AI, monitoring methods from non‐AI source’* *‘I'd want monitoring, quality assurance and transparency over processes to be in place so that I know what is AI looking for, how it works’* *‘I'd want to know if AI is being used and how it works, what is it doing and how it is used in decisions being made and influencing care’* *‘Important to know when it's being used, it needs full transparency for patients’* *‘Honesty is important and, to feel confident, I'd like to know where it is used and how it had been trained or developed’*	*‘Needs validation and pilots to build trust in use of AI’* *‘I am resistant to AI being too involved in provision of care and replacing dietitians’* *‘Research and knowing that other teams are using it would increase trust’* *‘Knowing its being used elsewhere and it has been piloted by comparing outcomes of AI use with regular practice’* *‘Important that patient knows it's an AI triage that is making decision about their care. It may require reassurance of dietitian involvement in the process or a written letter to explain what process it is, how decisions are made and contact details of teams to address concerns’* *‘Hope teams would be available to review and update the AI as is needed’* *‘Unsure if data privacy would be a concern but this could be due to limited knowledge of AI’*

#### 3.1.1 Theme 1: Potential Benefits of AI in Healthcare

Patients reported that AI in healthcare could empower clinician–patient interaction, accelerate triage process, facilitate decision‐making by reducing human error and biases and as a supporting tool in dietetics (e.g., design of meal plans, recipes). These views were echoed by dietitians who reported the benefits of AI in increasing the accuracy of diagnostic tools, dietary assessment, vetting process of referrals, triage decisions (i.e., plus alert for red flag symptoms) while they further emphasised its use in storing large amounts of data in one place and in administrative tasks to save dietitian time.

#### 3.1.2 Theme 2: Concerns about the Use of AI in Healthcare

It was emphasised by patients and dietitians that AI is not well‐established or well‐researched and it should be used with caution. Its use should not replace human interaction and reasoning, particularly with regards to care implementation and decision‐making at critical timepoints in clinical pathways or cases.

#### 3.1.3 Theme 3: Criteria When Applying AI in Healthcare

Both patients and dietitians reported that their trust to use AI in healthcare would increase if certain criteria were applied. These included the use of AI tools that are evidence‐based, validated and follow national guidelines or best practice, quality assurance and transparency over their use and continuous monitoring from governance bodies and non‐AI sources. Dietitians further reported that they would be more confident to use AI if other healthcare services and clinical teams were using it and provided full disclosure to patients where and how AI is used alongside the dietitian's role in decision‐making processes.

## 4 Discussion

This service evaluation has shown that integration of digital patient‐generated health data and patient‐reported outcomes into a triage process has more than doubled the clinical capacity of the triage clinic, reduced waiting times by over threefold and almost halved clinician time spent obtaining and analysing the information. It further improves patient‐centred care by providing dietitians with the information needed to allocate patients to appropriate dietary treatment (first‐ or second‐line advice) and mode of dietary education (group or one‐to‐one) for IBS management in a more timely manner. Furthermore, it demonstrated areas that AI could be used in triage and treatment pathways from a patient and clinician perspective.

Digital triage clinics provide an efficient process to gather relevant clinical information compared to telephone triage, which can be time‐consuming and an inefficient use of clinician time [38]. Digital triage has been shown to be timesaving and one community service reported that digital technology in triage has reduced waiting times for general practitioner appointments by 73% alongside improvements in clinical capacity without the need for additional general practitioner posts [39]. The data presented here support these findings and highlight the importance of the modernisation of the triage process in the dietetic pathway for improving service efficiency.

Maximising clinical capacity is vital in any healthcare service where it may not be feasible to obtain funding for new posts. Capacity and demand data in primary care can be used as examples where digital transformation of services increases clinical capacity [40].

Capturing the experiences and perspectives of stakeholders when innovating healthcare services is vital to empower patients and enable the development of user‐friendly services that are patient‐centred and meet the needs of individuals [41, 42, 43]. By using EBCD to develop the digital questionnaire, patients reported that the change from telephone to virtual triage was highly acceptable and the updated pathway allowed quicker access to dietary treatment and a chance to provide in‐depth details related to their clinical history. Valuing patient experience of healthcare in service development facilitates partnership working and identifies solutions to barriers and challenges not necessarily considered a priority by healthcare professionals [42].

The benefits of using a digital triage questionnaire can be applied to other specialties where services either use non‐digital triage to assess patients' suitability for group education or have large volumes of data to collect pre‐appointment. For patients who are directed to one‐to‐one appointments from digital triage, the data collected pre‐appointment helps to facilitate a patient‐centred appointment as more information is available to the clinician in advance than could be obtained verbally during the appointment time [3, 44]. There is a potential to apply this digital technology to other patient groups, for example those with coeliac disease, to help identify those patients who require one‐to‐one dietetic input at first diagnosis or annual review versus group education or digital resources. It is important to note that almost half of the patients during referral vetting went straight to a one‐to‐one appointment with the dietitian highlighting the need for continued access to one‐to‐one appointments as not all patients would be appropriate for this type of triage in a gastroenterology dietitian service.

The clinician's time wasted on patients who DNA'd appointments was four times higher during telephone triage before virtual triage implementation. However, incorporating a digital triage questionnaire into the IBS pathway limited access for 17% of patients who were unable to engage with the digital technology. Since implementing virtual triage, it has become standard practice to telephone patients if the questionnaire was not completed after 2 weeks. For most patients, this prompt enabled questionnaire completion. Where patients reported issues accessing the platform, the dietitian provided troubleshooting support; if unsuccessful, patients were redirected to non‐digital services. Where patients needed an interpreter or accessible services, appropriate support was offered. Patients who continued not to engage with the service without a reason were discharged. Health inequalities are common, and it is vital that alternative triage options are built into services to enable access for all [45]. Reduced access to services and lower patient‐reported outcomes have been observed in patients with IBS from lower socio‐economic or ethnic minorities groups [46], thus it is important to ensure that any digital transformation addresses health inequalities related to this [47].

This project is the first report of a digital questionnaire in the dietetic IBS pathway to include assessment of psychological stress and impact of IBS on daily life; other questionnaires have focused only on symptom assessment [26, 48]. Patients from the focus groups initially questioned why psychological stress and the impact of IBS on daily life were included in the questionnaire but on discussion with the specialist gastroenterology dietitians understood why they were relevant and appropriate text was carefully worded to be included at the start of this section. More recently, the need for a holistic approach to IBS care has taken centre stage, and dietitians are important members of the multi‐disciplinary team to discuss these challenges and provide information to patients on psychological treatment options available, for example, IBS‐specific cognitive behavioural therapy and gut‐directed hypnotherapy [49, 50].

There are several limitations to take into consideration. First, this implementation study was carried out in an urban inner‐city teaching hospital with a high volume of IBS referrals so it may not be generalisable for other services and therefore caution may need to be taken with reference to the impact on waiting times, clinical capacity and patient‐centred care. Second, the study took place at a time when a new electronic patient record (EPIC) was being incorporated into the direct healthcare of patients, which initially reduced clinical activity during 4‐weeks but in the longer term may have improved clinical efficiency for reasons beyond the researchers' control. Nevertheless, the pilot and implementation of the digital virtual triage reported here lasted for over 9 months after the launch of EPIC, allowing sufficient time to assess a clear improvement in waiting times and greater clinical capacity due to the integration of digital technology into an IBS pathway. Third, small and purpose sampling was used and may have been subjected to investigator and patient bias, for example, with respect to the EBCD of the digital questionnaire and the patient semi‐structured interviews, although patients from different socio‐economic backgrounds and ethnic groups were included.

The use of automation in identifying important answers, severe symptom responses or out‐of‐reference‐range results in the digital questionnaire was well received by the dietitians undertaking the virtual triage clinic as previously reported [44]. It helps to quickly identify variables that would indicate patients' unsuitability for group education. AI has been shown to benefit GP triage processes reducing waiting times and increasing clinical capacity [39] as well as improving the efficiency of the referral management or vetting pathway in gastroenterology services with significant time saved on reviewing GP referrals in secondary care [51]. Further development of the AI process could include development of the tool so that certain patients are automatically triaged to a one‐to‐one appointment rather than a group based on specific criteria. For example, if the patient reported weight loss and/or history of an eating disorder, they would automatically be directed to a one‐to‐one appointment with the dietitian rather than the dietitian needing to triage the referral taking up a virtual triage appointment slot, hence reducing the need to triage all referrals. The current study showed that a higher proportion of patients in secondary care than in primary care were unsuitable for triage and needed individualised appointments so this may be important to acknowledge when developing new healthcare services involving primary and secondary care referrals.

There is a possibility that AI decision support systems could be used within the digital triage process in dietetics but with AI still in its infancy within healthcare [52], it was insightful to understand the attitudes of dietitians and patients towards the use of AI for the whole dietetic pathway, not just triage. There are particular tasks in dietetics, for example, dietary intake tracking, identification of dietary/nutritional inadequacies that would benefit from AI and would be well accepted and welcomed within dietetic practice as reported here and elsewhere [44, 53]. All the dietitians selected faster referral vetting and triage as a benefit of AI in dietetics. The lack of empathy and understanding that AI can offer is recognised [8] and this was echoed by dietitians and patients alike showing the value of the patient–clinician relationship in IBS management. Administrative tasks can become a burden and contribute to clinician dissatisfaction or burnout [25]. The current study reported that using AI in administrative tasks may be acceptable given the minimal patient contact this involves but this process still requires validation, testing and staff upskilling before it can be safely adopted.

Staff in clinical roles are often reliant on the healthcare system's digital teams, clinical academics and researchers to drive forward digital innovations, such as the exploration of the use and integration of AI into electronic patient records. This is the type of work that builds confidence in the technology among clinical staff and has the potential benefit to help clinicians overcome challenges that they face [54].

Patient attitudes towards the use of AI in healthcare varied between the three patients who were interviewed; however, with a small cohort, it is not possible to assume all patients share the same apprehensions around AI. Attitudes towards to use of AI in dietetics seem to be positive but understandably tentative and again with a small number of dietitians interviewed may not reflect the views of the wider dietetic community. Without full understanding and demonstration of how AI can be safely incorporated into dietetic practice, there may be slow uptake of it as reported elsewhere [8, 44].

In conclusion, digital transformation of a telephone triage clinic to a virtual triage process has been shown to increase clinical capacity, reduce waiting times without increasing clinician burden. The EBCD component has demonstrated that a patient‐centred care approach leads to the development of an effective and highly efficient way of gathering clinical information to guide clinical decision making. Electronic health record integrated AI clinical decision support systems warrant further validation to increase confidence and acceptability for both clinicians and patients but certainly could help to solve problems and improve healthcare efficiency.

## Author Contributions

Miranda Lomer designed the digital triage implementation project, secured funding, supported focus groups, analysed the data, designed tables and figures and wrote the manuscript. Emma Stennett carried out patient and dietitian interviews, developed the digital questionnaire, collected data, analysed data, designed tables and figures and wrote the manuscript. Katerina Belogianni facilitated the design of the qualitative interviews and supported focus groups. All authors reviewed and commented on the final manuscript.

## Conflicts of Interest

Miranda Lomer leads post‐registration courses for dietitians on the dietary management of irritable bowel syndrome. The remaining authors declare no conflicts of interest.

## Supporting information


**Table S1:** Domains, construct and detailed application for use of the consolidated framework for implementation research (CFIR). **Table S2:** Telephone triage and virtual triage questions. **Table S3:** Semi‐structured interview questions on attitudes to AI use in healthcare. **Table S4:** Patient comments and responses for questionnaire development. **Box S1:** Virtual triage implementation detail. **Box S2:** External planned update of hospital electronic patient record.

## Data Availability

The data that support the findings of this study are available on request from the corresponding author. The data are not publicly available due to privacy or ethical restrictions.
