# Supplementary material for: Development and Implementation of Novel Virtual Triage and Exploration of Attitudes Towards the Potential Use of Artificial Intelligence in the Irritable Bowel Syndrome (IBS) Dietetic Pathway
Source: J Hum Nutr Diet. 2026 Feb 4;39(1):e70210. doi: 10.1111/jhn.70210 (PMC12872205; doi:10.1111/jhn.70210)
Supplement: Supplementary file 1 — Table S1: Domains, construct and detailed application for use of the consolidated framework for implementation research (CFIR). Table S2: Telephone triage and virtual triage questions. Table S3: Semi‐structured interview questions on attitudes to AI use in healthcare. Table S4: Patient comments and responses for questionnaire development. Box S1: Virtual triage implementation detail. Box S2: External planned update of hospital electronic patient record. [file JHN-39-0-s001.docx]

**Supporting information**

**Table S1: Domains, construct and detailed application for use of the consolidated framework for implementation research (CFIR)**

| **Domain** | **Construct** | **Application to virtual triage project** |
| --- | --- | --- |
| **Intervention characteristics** | Need of intervention | High volume of referrals, increased waiting lists post the COVID-19 pandemic, need for digitalisation, increase clinic capacity without an increase in staff resource |
|  | Core and peripheral components | Develop comprehensive triage questionnaire from a simple symptom assessment questionnaire  Questionnaire adapted to meet patient and clinic requirements |
|  | Trialability | Test the questionnaire in one clinical site and then incorporate it into clinical pathway |
| **Outer setting** | Patient needs and resources | Assessment of patient needs towards providing patient-centred care and guided by patient characteristics and symptoms |
|  |  | Consideration of barriers and facilitators to address the above needs |
|  | Policy | NHS moves and supports digitalisation |
| **Inner setting** | Structural characteristics | Compatibility of the new triage process with the current dietetic service process; staff involvement; facilitates communication within the organization and health professionals, promotes ‘team work’ |
| **Individual characteristics** | Knowledge and beliefs about the intervention | Experience based co-design captures attitudes toward and value placed on the intervention and self-efficacy to use the intervention |
| **Process** | Planning & engaging | Co-participatory approach |
|  | Execution | Implementation in real-life setting |
|  | Reflecting and evaluating | Number of people completing the questionnaires, difficulties encountered, evaluating outcomes |

**Table S2: Telephone triage and virtual triage questions**

| **Question topics** | | **Telephone triage** | | **Virtual triage** |
| --- | --- | --- | --- | --- |
|  |  | **Pre-appointment questionnaire** | **Telephone appointment** | **Digital questionnaire** |
| Symptom assessment | |  |  |  |
|  | Symptom history/presentation |  |  | ✓ |
|  | Global symptom question (1)^a^ | ✓ |  | ✓ |
|  | Gastrointestinal symptom rating scale (2)^a^ | ✓ |  | ✓ |
|  | Bristol stool form scale (3)^a.b^ | ✓ |  | ✓ |
|  | Stool frequency | ✓ |  | ✓ |
|  | IBS-SSS (4)^a,b^ |  |  | ✓ |
|  | Clinical concerns^c^ |  |  | ✓ |
| Nutritional assessment | |  |  |  |
|  | Anthropometrics (including recent weight loss) |  | ✓ | ✓ |
| Medical history | |  |  |  |
|  | Medical History |  | ✓ | ✓ |
|  | Surgical History |  |  | ✓ |
|  | Previous diagnosis (coeliac disease, inflammatory bowel disease, pancreatitis, diverticular disease, bile acid malabsorption, endometriosis, diabetes) |  |  | ✓ |
|  | Current/previous eating disorder or disordered eating |  | ✓ | ✓ |
| Relevant investigations | |  |  |  |
|  | Coeliac screen |  | ✓ | ✓ |
|  | OGD, colonoscopy, CT, MRI, ultrasound |  | ✓ | ✓ |
| Drug History | |  |  |  |
|  | Prescribed medication |  | ✓ | ✓ |
|  | Over the counter medication |  |  | ✓ |
| Dietary assessment | |  |  |  |
|  | Food allergies and intolerances |  |  | ✓ |
|  | Perceived dietary triggers, food avoidance, dietary restrictions |  | ✓ | ✓ |
|  | Dietary intake (food and fluid) |  | ✓ | ✓ |
|  | Alcohol intake (5) |  |  | ✓ |
|  | Fruit and vegetable intake^d^ |  |  | ✓ |
| Psychological impact questions | |  |  |  |
|  | Perceived stress scale (PSS) (6) |  |  | ✓ |
|  | Work & social adjustment scale (WSAS) (7) |  |  | ✓ |
| Equality, diversity & inclusion assessment | |  |  |  |
|  | Language barrier |  | ✓ | ^e^ |
|  | Learning disability |  | ✓ | ^e^ |

^a^ Validated tools, ^b^ May require a licence to use in clinical practice, ^c^ Clinical concerns include co-morbidities that require personalised dietary counselling, unexplained weight loss, blood in stool, unexplained anaemia, history of disordered eating or an eating disorder and scores for IBS-SSS >300, PSS >27 and WSAS > 20 ^d^ high in fermentable oligosaccharides, disaccharides, monosaccharides and polyols (FODMAPs) ^e^ identified via non-completion of questionnaire or administrative team contact necessitating one-to-one appointment

**Table S3 Semi-structured interview questions on attitudes to AI use in healthcare**

| **Questions and probes** | |
| --- | --- |
| Are you familiar with the concept of artificial intelligence (AI) and do you have any understanding or experience of its use in healthcare? | |
|  | *Patient probe – Have you experienced AI used in another clinical service?* |
|  | *Dietitian probe – Have you seen other clinical services use AI?* |
| How would you feel if AI were used to help determine the urgency of patient care needs? | |
|  | *Patient probe – How would you feel if AI was used to determine the outcome of your IBS triage* |
|  | *Dietitian probe – would you trust AI to determine the outcome of a patients IBS triage questionnaire* |
| In what ways do you think AI could help improve healthcare triage, such as identifying which patients need quicker care? Are there specific benefits you imagine? | |
| Do you have concerns about AI being used in healthcare decision-making? | |
|  | *Patient probe –How would you feel if AI made decisions about what treatment you needed* |
|  | *Dietitian probe – Would you trust AI to make decisions about your patients care needs?* |
| Would you feel comfortable if AI was involved in assessing patient information to help triaging to care? | |
| How much trust would you have in AI to make initial decisions about patient care? What could help increase your trust? | |
| Do you feel that an AI system could understand patient’s unique health needs as well as a human provider? | |
| Do you think that using AI to triage patients could affect the quality of care patients receive? If so, in what way? | |
| Do you think patients would want to know if AI was involved in their healthcare decisions? | |
| How important is it for patients to be informed about AI use in their healthcare? | |
| How do you feel about the potential for AI to be used more widely in healthcare? Do you think it will improve healthcare or lead to challenges? | |
| Would you prefer AI is used as a support tool, or as an independent decision-maker? | |
| What would help you feel more comfortable with AI being used in healthcare? | |
| Are there any other thoughts or concerns you’d like to share about how you feel regarding AI being used in healthcare? | |

**Table S4 Patient comments and responses for questionnaire development**

| **Patient focus group comments** | | **Actions taken** |
| --- | --- | --- |
| Ease of use of digital questionnaire | | |
|  | Difficult to complete by older people or non-tech persons | Identify barriers to completion and offer alternatives such as telephone triage or individual appointment if unable to engage digitally |
|  | Offering the option of paper version for non-tech people | Avoid unnecessary printing of questionnaire and offer alternative triage or individual appointment |
|  | It took longer than expected to complete, the link went to my junk folder. | Included completion time expectation in introduction.  Communication methods updated so patients receive text, email and letter correspondence and to check junk folder. |
|  | Easy to understand |  |
|  | Some terms were not so clear to understand, for example, it was difficult to describe stools | Language simplified or additional description/ visuals included to help with stool description |
| Preference for digital questionnaire vs telephone appointment with dietitian/dietetic assistant | | |
|  | Digital questionnaire offers a lot of time to complete it and think or change the answers | Patients advised they have 2 weeks to complete the questionnaire. Answers can be reviewed and edited before submission. |
|  | Prefer digital questionnaire to telephone triage |  |
|  | Option on each page to add more detail | Comments box included at the end of each section for patients to elaborate on answers |
|  | Digital form is a good direction and addition to the service, prefer this to telephone triage |  |
|  | Would like to receive a copy of questionnaire answers | Patients able to review questionnaire answers and access letters/ outcome of triage in online account to enable digital triage to be sustainable |
| Introduction information to describe the purpose of the questionnaire | | |
|  | Original text  ‘Your doctor has referred you to the Gastroenterology Dietitian for advice to help with your gut/bowel symptoms. We aim to provide you with practical, effective dietary advice that is appropriate for your symptoms and history.  To ensure we have enough information to do a thorough assessment, please complete the following history and symptom questionnaire to provide us with the information we need for our assessment. We will then be able to review your answers and ensure you are provided with suitable dietary advice.  If you are unable to complete and return the questionnaire electronically or require support with completion, then please contact us [xxx] and we will be able to make alternative arrangements.  Please complete this questionnaire within 2 weeks of receiving it. The results will enable us to plan what further management is appropriate for your history and symptoms. If you do not complete it then we will assume you no longer require input and you will be discharged back to the referring doctor.’ | Updated text  ‘You have been referred to the Gastroenterology Dietitians. As part of your assessment we request that you complete an online questionnaire about your symptoms and history so that we can identify the most appropriate management plan for you.  **What do you need to do next?**  Complete the questionnaire within two weeks of this letter being sent.  The questionnaire will ask about:   - Your gut (bowel) symptoms and how they affect your day-to-day life - Your medical, surgical and medication history - Your dietary habits   By completing this questionnaire, you agree that your information can be held on the electronic patient system and accessed by the staff involved in your care.  The questionnaire should only take about 20 minutes. We are unable to book you a further appointment if this questionnaire has not been completed. If you are unable to complete and return the questionnaire electronically or require support with completion, then please contact us [xxx] and we will be able to make alternative arrangements.  If you do not complete this questionnaire without informing us we may not make any further appointments for you and your treatment may be delayed.’ |
|  | Have some detail to explain what is needed i.e. medication, scans, dates | Introduction updated to inform patients of the content of questionnaire before it is started |
|  | If feels very threatening and negative to include ‘will be discharged’ if questionnaire not completed | Wording updated as above and uses your treatment may be delayed |
|  | Wording in the introduction to ‘if questionnaire not completed you will be discharged’ feels too harsh, sometimes things get missed and don’t want to feel in trouble. | Pathway updated to ensure patients get a telephone call if they have not completed the questionnaire after 2 weeks |
| Symptom information | | |
|  | Maybe add in the form ‘what do you think brought on your IBS?’ to get a perspective of why you have the symptoms, i.e., understand the triggers. | Question included ‘Do you think anything has caused your bowel/gut symptoms? (e.g. food poisoning, antibiotics, new mediations, surgery, stress etc., please give any relevant details)’ |
|  | Confused with the questions in relation to the severity and frequency of symptoms as these may vary from day to day | The questions are within validated questionnaires so it was agreed to add a comments box at the end of the section for patients to elaborate on symptom variation |
|  | Answer options are easy to complete: none, mild, moderate, severe |  |
|  | Sliding scale for IBS-SSS difficult on smartphone or tablet | Feedback sent to IT team to improve usability |
|  | Not all patients have typical ‘IBS’ | Changed reference from ‘IBS’ to ‘gut/ bowel symptoms’ where possible |
| Medical, surgical and diet history | | |
|  | Add option for unsure of dates of investigations | Free text box allows patients to comment if dates unknown |
|  | List of medical conditions – some maybe being investigated or suspected rather than having a confirmed diagnosis | Updated title to ‘Medical conditions you have been diagnosed with or suspect you have’ |
|  | I had hay fever as a child, but the option has the word ‘current’ or ‘tick if present’ so he found it confusing/ difficult to answer | Updated selected options to be ‘No, Yes/ current ongoing, Previously in adulthood, Previously in childhood, Do not know/ would like to discuss’ |
| Dietary assessment | | |
|  | Add in examples of portion sizes | Portion sizes of foods from that section added in as examples |
|  | Include caffeinated and decaffeinated drinks separately | Section for caffeinated or decaffeinated drinks added |
|  | Maybe ‘give more than one example’ is better to prompt people to write more examples | Updated question to ‘Please detail your normal breakfast intake, give a few examples of what you tend to have throughout the week’ |
|  | Has irregular fruit and vegetable intake, so it feels difficult to make a precise estimation of intake | Added in portion size guide/ suggestions to help patients answer question |
|  | Add in prompts for volume of fluid / examples of sizes | Added in typical volumes/ standard volumes to each fluid section ‘3x 500mls bottles, number of glasses per day/ 1 large glass of lemonade, 1 can cola per day’ |
| Alcohol | |  |
|  | Easy to complete/ understand |  |
|  | Good to have option to not complete if don’t feel comfortable sharing | Included ‘Are you happy to provide details on your alcohol intake?’ at start of section. |
| Perceived stress and work and social and work adjustment scale | | |
|  | IBS affects all of this and is a constant thing so good to be acknowledged |  |
|  | I am reluctant to talk to doctors about ‘hard’ life events because everyone attributes everything to stress. Maybe helpful to reassure people that these questions are asked because there are ways to help | Updated introduction to this section to  ‘A hectic lifestyle, anxiety and stress can all affect the gut, which in turn may increase symptoms. We would like to know if there are any of these aspects that might be affecting you, so that we can direct you to the most appropriate support.’ |
|  | Easy to understand why these sections are here |  |
|  | Easy to understand why these questions were being asked. The questions are self-reflective, and the topic/subjects are delicate |  |
| What happens next section | |  |
|  | Original text  ‘Thank you for completing this questionnaire  This questionnaire will be reviewed. If we need further information, we will contact you by telephone, otherwise you will be provided with an appointment to provide you with a management plan most appropriate for you.’ | Updated text  *‘*Thank you for completing this questionnaire.  The questionnaire will be reviewed by a gastroenterology dietitian within two weeks of you submitting your response. Usually you will NOT speak to the dietitian at this point unless more information is needed. Once your questionnaire answers have been reviewed you will receive a letter to explain the next steps in your dietary management plan based on your answers.*’* |
|  | Inform about the waiting time until the appointment | Unable to include as waiting times vary depending on pathway |
|  | Provide a timeline to hear back | Updated information to say questionnaire will be assessed within 2 weeks of submission |
|  | Say thank you this will be reviewed by a clinician, reassure that someone appropriate/ a real human will review the forms as these things are quite personal | Updated information to say ‘This questionnaire will be reviewed by a member of the gastroenterology team’ and ‘Thank you for taking the time to complete this questionnaire’ |

**Box S1: Virtual triage implementation detail**

From August 2023, patients vetted as suitable for triage were added to the waiting list for the virtual triage clinic. Patients unsuitable for virtual triage included those previously described unsuitable for telephone triage (i.e. patients unsuitable for group education, see referral vetting in methods for further detail) and those who reported a lack of digital literacy were offered one-to-one appointments. A lack of digital literacy was where patients reported they were not competent to access the digital platform or able to use or access the internet.

Suitable patients were sent a letter and a text message with a prompt to complete a digital questionnaire within 2 weeks with reminders at one week and 10 days later if they had not already completed the questionnaire. The administrative team contact details (email and telephone number) were provided to support patients who were unable to complete the questionnaire. The administrative team sought advice from the virtual triage clinic dietitian to determine what type of appointment the patient should be booked into if the questionnaire could not be completed.

Completed questionnaires were reviewed by the dietitian responsible for the virtual triage clinic. Patients were contacted by telephone if the dietitian needed further information related to any responses that were of clinical concern. In addition, patients whose questionnaire indicated symptom resolution were provided with a telephone call to determine if they still needed dietary interventions, if not they were discharged from the IBS pathway. Patients who had not completed the questionnaire also received a telephone call to troubleshoot reasons for non-completion. If patients were unavailable by telephone, one further reminder was sent requesting patients to complete the questionnaire indicating their treatment may be delayed if they did not.

The outcome of virtual triage was provided to patients via text and/or letter. The outcome decisions were one of the following: 1) one-to-one appointment 2) online group education on: first-line dietary advice for IBS, dietary advice for constipation or the low FODMAP diet or 3) discharge from service for patients no longer needing dietary management. Patients who did not complete the digital questionnaire following all reminders were discharged from the service with an opportunity to rebook within 2 weeks if they still needed dietary management.

**Box S2: External planned update of hospital electronic patient record**

A planned update of the hospital electronic patient record took place in October 2023 to a single system called EPIC Healthcare (EPIC systems corporation, [Verona, Wisconsin](https://www.bing.com/ck/a?!&&p=0b9c4de4dd54f1cefa9dca71bf294d49e8f1ecc7719e791e1238fbb7e3b1b74bJmltdHM9MTczMTk3NDQwMA&ptn=3&ver=2&hsh=4&fclid=340f0857-d065-6085-2d60-1ca4d18561a8&u=a1L3NlYXJjaD9GT1JNPVNOQVBTVCZxPVZlcm9uYSUyYytXaXNjb25zaW4mZmlsdGVycz1zaWQ6ImEzOGM2NThmLTVmMGMtYTc5ZS01YmU1LTAyMmVlMWE3NjhjYSI&ntb=1), USA). EPIC delivers and stores all information about patients’ care in one place and works alongside an associated patient facing app called MyChart (EPIC systems corporation, [Verona, Wisconsin](https://www.bing.com/ck/a?!&&p=0b9c4de4dd54f1cefa9dca71bf294d49e8f1ecc7719e791e1238fbb7e3b1b74bJmltdHM9MTczMTk3NDQwMA&ptn=3&ver=2&hsh=4&fclid=340f0857-d065-6085-2d60-1ca4d18561a8&u=a1L3NlYXJjaD9GT1JNPVNOQVBTVCZxPVZlcm9uYSUyYytXaXNjb25zaW4mZmlsdGVycz1zaWQ6ImEzOGM2NThmLTVmMGMtYTc5ZS01YmU1LTAyMmVlMWE3NjhjYSI&ntb=1), USA). During October 2023, there was a planned increase in waiting times for appointments while EPIC and MyChart were adopted with some initial delays with patients not receiving digital communication, which was soon rectified.

**Supporting information references**

1. Mangel AW, Hahn BA, Heath AT, Northcutt AR, Kong S, Dukes GE, et al. Adequate relief as an endpoint in clinical trials in irritable bowel syndrome. J Int Med Res. 1998;26(2):76-81.

2. Svedlund J, Sjodin I, Dotevall G. GSRS--a clinical rating scale for gastrointestinal symptoms in patients with irritable bowel syndrome and peptic ulcer disease. Dig Dis Sci. 1988;33(2):129-34.

3. Lewis SJ, Heaton KW. Stool form scale as a useful guide to intestinal transit time. Scand J Gastroenterol. 1997;32(9):920-4.

4. Francis CY, Morris J, Whorwell PJ. The irritable bowel severity scoring system: a simple method of monitoring irritable bowel syndrome and its progress. Aliment Pharmacol Ther. 1997;11(2):395-402.

5. Bradley KA, DeBenedetti AF, Volk RJ, Williams EC, Frank D, Kivlahan DR. AUDIT-C as a brief screen for alcohol misuse in primary care. Alcohol Clin Exp Res. 2007;31(7):1208-17.

6. Cohen S, Kamarck T, Mermelstein R. A global measure of perceived stress. J Health Soc Behav. 1983;24(4):385-96.

7. Mundt JC, Marks IM, Shear MK, Greist JH. The Work and Social Adjustment Scale: a simple measure of impairment in functioning. Br J Psychiatry. 2002;180:461-4.
